# The CuePed Trial: How Does Environmental Complexity Impact Cue Effectiveness? A Comparison of Tonic and Phasic Visual Cueing in Simple and Complex Environments in a Parkinson's Disease Population with Freezing of Gait

**DOI:** 10.1155/2019/2478980

**Published:** 2019-07-24

**Authors:** Rodney Marsh, Michael H. Cole, Nadeeka N. W. Dissanayaka, Tiffany R. Au, Sandra Clewett, John D. O'Sullivan, Peter A. Silburn

**Affiliations:** ^1^School of Medicine, University of Queensland, Royal Brisbane & Women's Hospital, Herston, QLD 4029, Brisbane, Australia; ^2^Mental Health Service, Royal Brisbane & Women's Hospital, Herston, QLD 4029, Brisbane, Australia; ^3^School of Behavioural and Health Sciences, Australian Catholic University, Brisbane Campus, 1100 Nudgee Road, Banyo, QLD 4014, Brisbane, Australia; ^4^University of Queensland, UQ Centre for Clinical Research, Building 71/918 Royal Brisbane & Women's Hospital, Herston, QLD 4029, Brisbane, Australia; ^5^Neurology Research Centre, Level 7, Ned Hanlon Building, Royal Brisbane & Women's Hospital, Herston, QLD 4029, Brisbane, Australia; ^6^School of Health & Rehabilitation Sciences, University of Queensland, St Lucia, QLD 4067, Brisbane, Australia; ^7^Queensland Brain Institute, St Lucia, QLD 4067, Brisbane, Australia

## Abstract

**Background:**

The optimal prescription of cueing for the treatment of freezing of gait (FoG) in Parkinson's disease (PD) is currently a difficult problem for clinicians due to the heterogeneity of cueing modalities, devices, and the limited comparative trial evidence. There has been a rise in the development of motion-sensitive, wearable cueing devices for the treatment of FoG in PD. These devices generally produce cues after signature gait or electroencephalographic antecedents of FoG episodes are detected (phasic cues). It is not known whether these devices offer benefit over simple (tonic) cueing devices.

**Methods:**

We assembled 20 participants with PD and FoG and familiarized them with a belt-worn, laser-light cueing device (Agilitas™). The device was designed with 2 cueing modalities—gait-dependent or “phasic” cueing and gait-independent or “tonic” cueing. Participants used the device sequentially in the off, phasic, or tonic modes, across 2 tasks—a 2-minute walk and an obstacle course.

**Results:**

A significant improvement in mean distance walked during the 2-minute walk test was observed for the tonic mode (127.3 m) compared with the off (111.4 m) and phasic (116.1 m) conditions. In contrast, there was a nonsignificant trend toward improvement in FoG frequency, duration, and course time when the device was switched from off to tonic and to phasic modes for the obstacle course.

**Conclusions:**

Parkinson's disease patients with FoG demonstrated an improvement in distance walked during the two-minute walk test when a cueing device was switched from off to phasic and to tonic modes of operation. However, this benefit was lost when patients negotiated an obstacle course.

## 1. Introduction

Freezing of gait (FoG) is a common problem in people with Parkinson's disease (PD) and affects up to 87% of patients who have lived with the disease for over 10 years [1]. Whilst PD is a complex, multisystem disorder, FoG has been reported to have a greater impact on quality of life than any other symptom [2]. As the most common cause of falls in PD, FoG can have serious implications for patient morbidity, mortality, and quality of life. These implications have broader health economics consequences.

Current treatments for FoG generally involve manipulation of daily levodopa dose and timing, coupled with exercise and physiotherapy. There is also promising evidence for amantadine, methylphenidate, and subthalamic nucleus stimulation for the management of FoG, as well as case report level evidence for serotonin and norepinephrine reuptake inhibitors (SNRIs) [3, 4]. The clinical benefit from these interventions is often limited, and a clear need exists for further research aimed at establishing the efficacy of alternate methods of FoG management.

Cueing has long been recognized as a remarkably effective treatment in some patients with FoG [5]. However, given the complex neurobiology of FoG, each patient may respond differently to different cue modalities (e.g., visual, auditory, somatosensory, or cognitive) [6]. To date, there are no established predictors of patient responsiveness to a specific cueing strategy. In a recent meta-analysis that compared visual, auditory, and somatosensory cueing modalities, it was found that all three sensory modalities were comparably effective in a laboratory environment [6]. In contrast, an experimental study reported that visual cues were superior to auditory and vibration cues at assisting people with PD who had difficulties with gait initiation [7]. Unfortunately, the cues delivered in these studies were either (i) fixed, (ii) used a predetermined pulse, or (iii) voluntarily patient triggered. As such, much less is known about the efficacy of motion-triggered (phasic) cues for managing symptoms of FoG in people with PD. Ginis et al. [8, 9] have identified that there are difficulties with the long-term consolidation and transfer of the effects of cueing and further explored the possibilities that exist with advancing technologies, for the management of FoG with external cueing.

The capacity for miniaturization of electronic componentry has spawned the rapid development of a new generation of patient-worn devices, which may be used as cueing devices for the treatment of FoG [10–17]. A range of algorithms for the detection of FoG and the provision of gait-dependent cues are now in the public domain, and the Bachlin–Moore algorithm continues to be improved [10, 12]. In a recent meta-analysis of 23 studies [18], it was shown that studies seeking to detect FoG episodes using wearable sensors were highly variable with respect to the body part used to detect the events. There was also a significant degree of heterogeneity in the mode of cue delivery between studies, with an increasingly complex matrix of design options now available (e.g., modality, pulsed vs. continuous, patient vs. gait-initiated, and mechanical-aid associated). Collectively, these variables have made it difficult to determine the transferability of the reported outcomes to the real-world environment. To progress this field, there is a clear need for head to head comparative studies, where cue modality and/or environment is manipulated, to better understand the utility of cueing devices in all their forms.

The prescription of wearable devices for invivo use remains a significant problem. It is, however, a laudable goal in the knowledge that symptoms of FoG are generally most troublesome for patients in their home environments [15].

While the field continues to move apace, fundamental questions regarding the optimal prescription of cues in specific environments need to be answered. Importantly, we are unaware of how the newer motion-dependent technologies are compared with older technologies in simple versus complex environments. Will the quest for smarter, wearable cueing devices create a treatment that is of any more use than the inexpensive technologies that already exist? To begin to address this question, we designed a laboratory-based experiment with contrasting environments (simple and complex) to test the effectiveness of two different visual cueing modalities provided by a belt-worn cueing device worn by a PD population with FoG.

## 2. Method

A case series of 20 people with PD who were assessed by 3 local movement disorder neurologists in Brisbane, Australia, were included in the study. Patients were invited to participate in the study, if they were determined by their treating neurologist to have clinically significant FoG, and all participants reported a score ≥3 on item 3 of the Freezing of Gait Questionnaire [19] (Table 1). Participants were excluded if they had (i) a significant medical comorbidity that compromised their mobility; (ii) any visual impairment not corrected with lenses; or (iii) any significant cognitive impairment (Mini Mental State Exam total score <25). The study's protocol was registered with Clinicaltrials.gov (NCT02356536) and approved by the Human Research Ethics Committees at the three Brisbane-based hospitals involved in the trial. All volunteers provided written informed consent in accordance with the Declaration of Helsinki.

**Figure 1:**
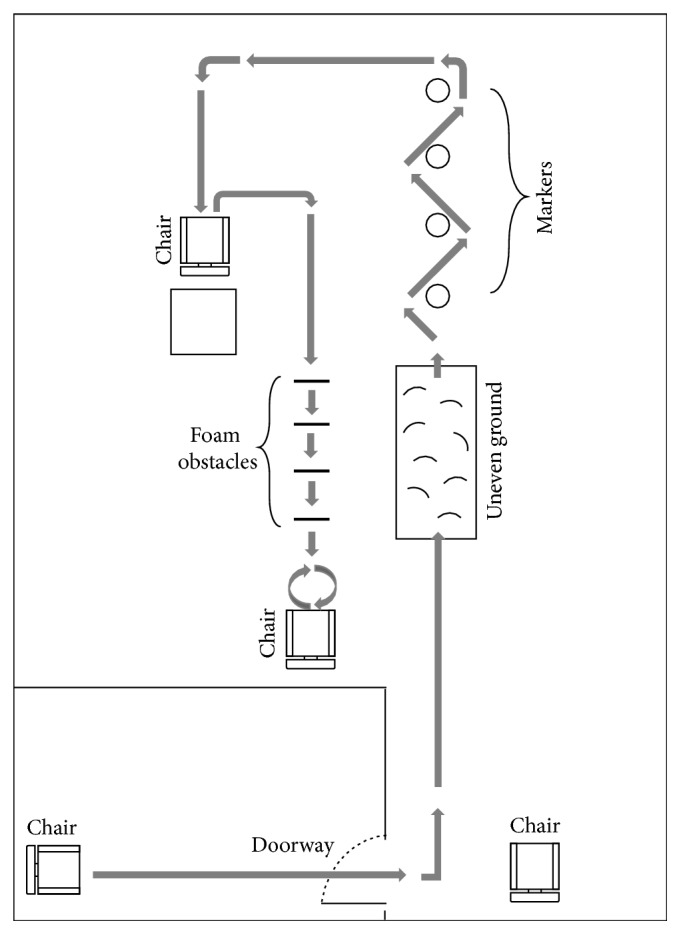
Schematic drawing of the 33-metre obstacle course.

Eligible participants completed the Freezing of Gait Questionnaire [19] to establish the frequency and impact of their FoG symptoms, while the motor subscale of the Unified Parkinson's Disease Rating Scale (UPDRS-III) was used to determine the severity of their motor symptoms. Following the assessments of symptom severity, participants were asked to perform 2 walking tasks that included (i) the 2-minute walk test (2MWT) and (ii) an obstacle course (Figure 1). The obstacle course involved standing from a seated position, walking 7 metres to an open doorway. After passing through the doorway, participants turned left and traversed an uneven walking surface, before weaving between four markers situated on the floor at 2-metre intervals. Once the final marker had been passed, participants turned left and made their way to a seat to sit down. Upon resting their back against the backrest of the seat, participants were asked to stand, turn 180° to their right, and walk towards a chair situated 10 metres away, at the other end of the room. While walking to the chair, participants were required to step over 4 foam obstacles that stood 0.15 metres tall and 1 metre apart. Before sitting, participants completed a full 360° turn in each direction.

**Figure 2:**
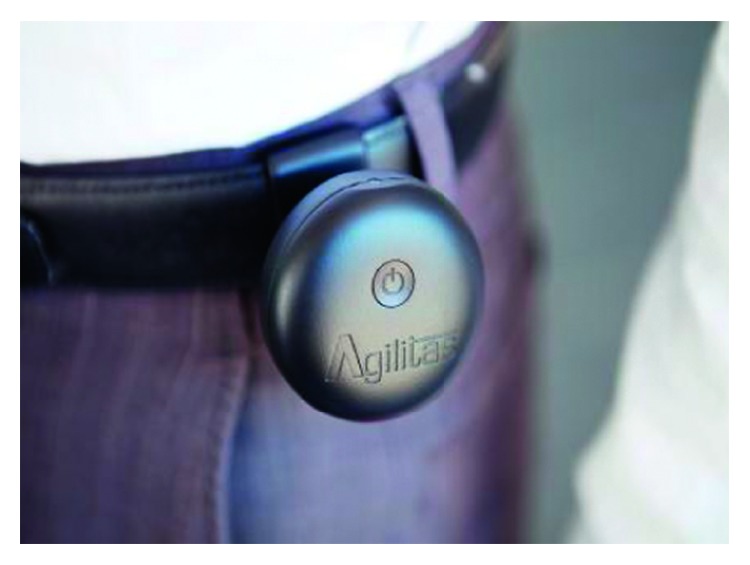
The belt-worn freeze-detecting visual cueing device. Note: the image shows the pilot light pointing upwards. By turning the device, the orientation of the visual cue can be personalised for each individual.

While performing each of these tests, participants wore a small belt-mounted device that was designed to detect the onset of FoG in people with PD using a series of incorporated microsensors (Figure 2). Specifically, this device used built-in accelerometers and gyroscopes sampling at 25 Hz to detect a series of signature kinematic patterns that are known to be antecedents of FoG episodes. When the device detected a kinematic pattern that was indicative of a gait disruption that would typically precede a FoG episode, an incorporated red-light laser pointer was triggered and projected a red dot several metres in front of the participant. While the light was active, the device continued to analyse the data from the in-built sensors and once the data suggested the resumption of steady state walking for at least 3 seconds, the light was extinguished, unless further triggered. To limit the risk of the device not providing a visual cue when an actual freezing episode occurred (i.e., false negatives), the FoG detection algorithm was deliberately calibrated to favour false positives (i.e., illuminating for complete and near FoG episodes). In addition to the FoG detection mode (i.e., the gait-dependent or “phasic” mode), it was also possible to set the laser pointer to provide a continuous or “tonic” visual cue or to switch it off. To evaluate the efficacy of the visual cueing device and to objectively determine the differences between phasic and tonic visual cueing modes, participants completed the two walking tasks for each of the 3 visual cueing modalities (i.e., off, phasic, and tonic). All trials were video recorded to assist with analysis, and to limit the potential influence of a learning effect and/or fatigue on the reported outcomes, the order of testing conditions was varied between participants. To ensure that the participants were optimally medicated at the time of testing, all procedures were undertaken within 1 to 2 hours of the participants' scheduled levodopa intake.

Following data collection, an associate investigator reviewed the video recordings of the 2MWT and the obstacle course on two occasions separated by at least one week. At each of these time points, the investigator identified the number and duration of FoG episodes experienced by each participant while performing the tasks with each of the visual cueing modalities. Statistical comparison of the two assessments indicated excellent intrarater reliability for the quantification of both the number (ICC: 0.930 to 0.975) and duration (ICC: 0.976 to 0.999) of FoG episodes for all visual cueing modes. In addition to recording the number and duration of freezing episodes, the distance covered by the participants during the 2MWT was also recorded in metres, while the time taken to complete the obstacle course was measured in seconds.

## 3. Statistical Analysis

The Shapiro–Wilk test was used to confirm that the primary outcomes were not normally distributed (*p* < 0.05) and supported the decision to use nonparametric statistical procedures. To statistically compare any mean differences between the off, phasic, and tonic visual cueing modalities for the frequency and duration of freezing episodes, the distance covered during the 2MWT, and/or the time taken to complete the obstacle course, the Friedman test was used. When a significant main effect was identified for cue type, pairwise comparisons were conducted with the Wilcoxon signed-rank test to further explore differences between the different visual cueing modalities. All statistical procedures were conducted using SPSS v.24, and the level of significance was set at *p* < 0.05.

## 4. Results

The results of the statistical analyses indicated that, while there was a gradient of improvement from the off to phasic and to tonic modes for all three measures, neither the frequency nor the duration of FoG episodes recorded during the 2MWT reached statistical significance (Table 2). However, a significant main effect was returned for distance walked by the patients during the 2MWT, with pairwise comparisons indicating that the participants walked further with the tonic visual cue compared with the off (*p*=0.026) and phasic (*p*=0.008) visual cue modalities.

While negotiating the obstacle course, there was no statistically significant improvement in FoG frequency (*p*=0.192), FoG duration (*p*=0.173), and course time (*p*=0.357) from the off condition to the tonic condition and the phasic condition (Table 3).

Whilst ultimately proving to be underpowered, due to the small differences in performance across modalities, the joint probability of the observed gradients of the 18 means across both courses was *p* < 0.001 (0.00002).

## 5. Discussion

Although FoG is a disabling and common problem in PD, there is a growing body of evidence for the benefits of cueing strategies in its treatment [6]. Specifically, previous research reports that the use of external cues can improve a range of gait parameters in PD patients, including gait speed, stride length, step variability, and cadence [20]. It is not known, however, whether cueing that is triggered in response to the specific kinematic events that precede the occurrence of a FoG episode is more effective than cueing that is fixed and independent of the FoG episode. Furthermore, it is not known how environment complexity may impact the effectiveness of these two cue modalities. While there is no accepted terminology for fixed versus motion-sensitive cues, we have chosen the terms “tonic” and “phasic” cueing as we believe these to be apt and widely understood terms that have historical neurophysiological meaning.

The results of our study showed that PD patients with FoG walked a greater distance during a 2-minute walk test with a tonic visual cue, compared with both the off (*p*=0.026) and phasic (*p*=0.008) cueing conditions. Because the distance walked improved but not the FoG duration and frequency, one explanation could be that the tonic availability of a visual cue for participants simply increased step amplitude and inhibited the sequence effect known to precede FoG. However, when subjects were asked to complete an obstacle course, there was a nonstatistically significant reduction of freezing episodes, freezing times, and course completion times when the device was switched from the off to tonic and to phasic modes.

Taken together, these results may suggest a superiority of a tonic cueing strategy in the simple environment of the 2-minute walk task but not in the complex environment of the obstacle course. While main effect measures and pairwise comparison of means were otherwise nonsignificant, it bears consideration that the differential gradients observed for every measure favoured tonic cueing during the simple 2MWT, while phasic cueing was better in the complex obstacle course. The reduced benefit of tonic cueing during the obstacle course could point to an influence of “environmental attention burden” on the cue's effectiveness and possibly shines further light on the pathophysiology of FoG and the mechanism of action of cueing.

There are currently four prevailing models that are used to understand the phenomenon of FoG [21]: (i) the threshold model; (ii) the neural reserve model; (iii) the cognitive model; and (iv) the decoupling model. However, the complex findings presented in this study do not specifically fit with any of these models and, hence, leads us to speculate that the central place of attention and attention regulation may be sufficiently important to warrant the proposition of a fifth distinct “Bayesian” model. There has been an increasing interest in conceptualizing neurological function and dysfunction through the lens of Bayes' theorem [22]. In the neurosciences, the approach has been useful, with the notion that the reconciliation of priors (that is, previously-encoded programs) with current data (that is, sensory input) can go awry. Attention acts as the modulator between these two domains, and it appears that the model fits with what is observed in FoG and may be supported by our findings.

Freezing occurs as an intermittent, dynamic process, precipitated by events thought to confer attentional cost to the subject, such as dual tasking, anxiety, or turning. It arises in a setting where there is already a loss of gait automaticity. Our findings pose the question of whether the disruption of misplaced attention brought on by cueing somehow facilitates a return to automaticity or a cortical takeover of the movement as suggested by Plotnik et al. [23].

Recent work utilizing virtual reality paradigms and fMRI scanning in simulated FoG shows an impaired “change” activation in the pre-supplementary motor area (SMA) region purported to be due to reduced feed-forward processing [24]. Circumstances requiring internally driven motor control (priors) are known to utilize bottom-up, dorsal visual pathways, described as covert attention. It is suggested that it is this covert attention that requires support and that it may be plausible that visual cueing's mechanism of action is through supporting this system.

Although untestable in a moving patient, it seems likely that tonic cueing may provide more optimal attention network and pre-SMA support in a simple environment and facilitate a return to automaticity and motor priors. In a more complex environment, such as an obstacle course with greater attentional demands, attention must be made available for current environmental sensory data in preference to motor priors. As such, a tonic visual cue would not suffice in this setting and another mechanism would be needed.

Plotnik et al. [23] suggested that a cortical takeover of movement occurs with cueing. The question arises as to whether a cortical takeover or a return to automaticity might predominate and whether this is dependent on the attention burden of the environment being negotiated.

## 6. Conclusions and Future Directions

This study suggests a superiority of tonic visual cueing over phasic or no visual cueing in a PD population with FoG when performing a 2-minute walk test in a simple environment. However, this finding was not maintained in the complex environment of an obstacle course in the same population. A nonsignificant differential gradient of improvement of all measures favouring tonic cueing in a simple environment and phasic cueing in a complex environment was observed. This may have implications for the use of visual cueing as a treatment for FoG in PD populations.

Further research is needed to consolidate this study's findings and determine whether there is benefit of phasic cueing over both continuous and pulsed tonic cueing. Furthermore, there is a clear need for studies to examine the effectiveness of patient-worn cueing devices for a longer duration of time, in a home environment, where freezing is often worse.

For this to be achievable, beyond the detection of antecedents of an impending freeze, the device would need to be capable of reliably detecting and measuring the FoG episodes themselves, to meaningfully function as a remote patient monitoring device. Given the heterogeneity of FoG, this task represents a significant challenge but is an exciting prospect in the treatment of these symptoms.

## Figures and Tables

**Table 1:** Patient characteristics.

	Mean (frequency)	SD (% sample)
Age	70.1	7.2
Gender (male)	15	75
UPDRS-III	36.4	13.5
Falls Efficacy Scale	34.8	12.8
Freezing of Gait Questionnaire	14.3	4.4
Montreal Cognitive Assessment	26.5	2.5
Standardised Mini Mental State Examination	28.4	1.2

**Table 2:** The frequency and duration of freezing episodes and the distance walked by the participants during the 2MWT completed under the off, tonic, and phasic visual cue conditions. Data represent means and standard deviations.

	Visual cue modality			Main effect	Pairwise comparisons		
	Off	Tonic	Phasic	*p* value	Off vs. tonic	Off vs. phasic	Tonic vs. phasic
FoG frequency (*n*)	1.11 (2.23)	0.95 (1.65)	1.06 (1.71)	0.459			
FoG duration (s)	5.42 (11.82)	2.68 (5.61)	3.47 (5.75)	0.114			
Distance (m)	111.44 (80.51)	127.30 (87.06)	116.08 (81.89)	0.014	0.026	0.394	0.008

*Note.* Off = no visual cue; tonic = continuous visual cue (i.e., gait-independent); phasic = FoG-sensitive visual cue (i.e., gait-dependent).

**Table 3:** The frequency and duration of freezing episodes and the time taken to complete the obstacle course under the off, tonic, and phasic visual cue conditions. Data represent means and standard deviations.

	Visual cue modality			Main effect	Pairwise comparisons		
	Off	Tonic	Phasic	*p* value	Off vs. tonic	Off vs. phasic	Tonic vs. phasic
FoG frequency (*n*)	2.88 (2.69)	1.82 (2.24)	1.71 (1.57)	0.192			
FoG duration (s)	28.88 (43.91)	18.41 (48.41)	15.35 (36.07)	0.173			
Course time (s)	99.76 (51.83)	82.65 (55.12)	81.06 (43.07)	0.357			

*Note.* Off = no visual cue; tonic = continuous visual cue (i.e., gait-independent); phasic = FoG-sensitive visual cue (i.e., gait-dependent).

## Data Availability

The data used to support the findings of this study are available from the corresponding author upon request.

## References

[1] Auyeung M., Tsoi T. H., Mok V. (2012). Ten year survival and outcomes in a prospective cohort of new onset Chinese Parkinson’s disease patients. *Journal of Neurology, Neurosurgery & Psychiatry*.

[2] Walton C. C., Shine J. M., Hall J. M. (2015). The major impact of freezing of gait on quality of life in Parkinson’s disease. *Journal of Neurology*.

[3] Devos D., Moreau C., Delval A., Dujardin K., Defebvre L., Bordet R. (2013). Methylphenidate. *CNS Drugs*.

[4] Nonnekes J., Snijders A. H., Nutt J. G., Deuschl G., Giladi N., Bloem B. R. (2015). Freezing of gait: a practical approach to management. *The Lancet Neurology*.

[5] Dunne J. W., Hankey G. J., Edis R. H. (1987). Parkinsonism: upturned walking stick as an aid to locomotion. *Archives of Physical Medicine and Rehabilitation*.

[6] Rocha P. A., Porfírio G. M., Ferraz H. B., Trevisani V. F. M. (2014). Effects of external cues on gait parameters of Parkinson’s disease patients: a systematic review. *Clinical Neurology and Neurosurgery*.

[7] McCandless P. J., Evans B. J., Janssen J., Selfe J., Churchill A., Richards J. (2016). Effect of three cueing devices for people with Parkinson’s disease with gait initiation difficulties. *Gait & Posture*.

[8] Ginis P., Nackaerts E., Nieuwboer A., Heremans E. (2017). Cueing for people with Parkinson’s disease with freezing of gait: a narrative review of the state-of-the-art and novel perspectives. *Annals of Physical and Rehabilitation Medicine*.

[9] Ginis P., Heremans E., Ferrari A., Bekkers E. M. J., Canning C. G., Nieuwboer A. (2017). External input for gait in people with Parkinson’s disease with and without freezing of gait: one size does not fit all. *Journal of Neurology*.

[10] Bachlin M., Plotnik M., Roggen D. (2010). Wearable assistant for Parkinson’s disease patients with the freezing of gait symptom. *IEEE Transactions on Information Technology in Biomedicine*.

[11] Capecci M., Pepa L., Verdini F., Ceravolo M. G. (2016). A smartphone-based architecture to detect and quantify freezing of gait in Parkinson’s disease. *Gait & Posture*.

[12] Djuric-Jovicic M. D., Jovicic N. S., Radovanovic S. M., Stankovic I. D., Popovic M. B., Kostic V. S. (2014). Automatic identification and classification of freezing of gait episodes in Parkinson’s disease patients. *IEEE Transactions on Neural Systems and Rehabilitation Engineering*.

[13] Ferraye M. U., Fraix V., Pollak P., Bloem B. R., Debû B. (2016). The laser-shoe: a new form of continuous ambulatory cueing for patients with Parkinson’s disease. *Parkinsonism & Related Disorders*.

[14] Pilleri M., Weis L., Zabeo L. (2015). Overground robot assisted gait trainer for the treatment of drug-resistant freezing of gait in Parkinson disease. *Journal of the Neurological Sciences*.

[15] Rodriguez-Martin D., Pérez-López C., Samà A. (2017). A waist-worn inertial measurement unit for long-term monitoring of Parkinson’s disease patients. *Sensors*.

[16] Zhao Y., Nonnekes J., Storcken E. J. M. (2016). Feasibility of external rhythmic cueing with the google glass for improving gait in people with Parkinson’s disease. *Journal of Neurology*.

[17] Ahn D., Chung H., Lee H.-W. (2017). Smart gait-aid glasses for Parkinson’s disease patients. *IEEE Transactions on Biomedical Engineering*.

[18] Silva de Lima A. L., Evers L. J. W., Hahn T. (2017). Freezing of gait and fall detection in Parkinson’s disease using wearable sensors: a systematic review. *Journal of Neurology*.

[19] Giladi N., Treves T. A., Simon E. S. (2001). Freezing of gait in patients with advanced Parkinson’s disease. *Journal of Neural Transmission*.

[20] Djuric-Jovicic M., Jovicic N., Radovanovic S., Kresojevic N., Kostic V., Popovic M. (2014). Quantitative and qualitative gait assessments in Parkinson’s disease patients. *Vojnosanitetski Pregled*.

[21] Vercruysse S., Gilat M., Shine J. M., Heremans E., Lewis S., Nieuwboer A. (2014). Freezing beyond gait in Parkinson’s disease: a review of current neurobehavioral evidence. *Neuroscience & Biobehavioral Reviews*.

[22] Edwards M. J., Adams R. A., Brown H., Parees I., Friston K. J. (2012). A bayesian account of “hysteria”. *Brain*.

[23] Plotnik M., Shema S., Dorfman M. (2014). A motor learning-based intervention to ameliorate freezing of gait in subjects with Parkinson’s disease. *Journal of Neurology*.

[24] van der Hoorn A., Renken R. J., Leenders K. L., de Jong B. M. (2014). Parkinson-related changes of activation in visuomotor brain regions during perceived forward self-motion. *PLoS One*.

